# The effect of different sources of fish and camelina sativa oil on immune cell and adipose tissue mRNA expression in subjects with abnormal fasting glucose metabolism: a randomized controlled trial

**DOI:** 10.1038/s41387-018-0069-2

**Published:** 2019-01-09

**Authors:** Vanessa D. de Mello, Ingrid Dahlman, Maria Lankinen, Sudhir Kurl, Leena Pitkänen, David E. Laaksonen, Ursula S. Schwab, Arja T. Erkkilä

**Affiliations:** 1 Institute of Public Health and Clinical Nutrition, Faculty of Health Sciences, University of Eastern Finland, Kuopio, Finland; 2 Department of Medicine, Huddinge, Karolinska Institutet, Stockholm, Sweden; 3 Department of Ophthalmology, Institute of Clinical Medicine, University of Eastern Finland, Kuopio, Finland; 4 Department of Ophthalmology, Kuopio University Hospital, Kuopio, Finland; 5 Department of Medicine, Endocrinology and Clinical Nutrition, Kuopio University Hospital, Kuopio, Finland; 6 Institute of Biomedicine, Physiology, University of Eastern Finland, Kuopio, Finland

## Abstract

**Background/Objectives:**

Molecular mechanisms linking fish and vegetable oil intakes to their healthy metabolic effects may involve attenuation of inflammation. Our primary aim was to examine in a randomized controlled setting whether diets enriched in fatty fish (FF), lean fish (LF) or ALA-rich camelina sativa oil (CSO) differ in their effects on the mRNA expression response of selected inflammation-related genes in peripheral blood mononuclear cells (PBMCs) and subcutaneous adipose tissue (SAT) in subjects with impaired fasting glucose.

**Subjects/Methods:**

Samples from 72 participants randomized to one of the following 12-week intervention groups, FF (*n* = 19), LF (*n* = 19), CSO (*n* = 17) or a control group (*n* = 17), were available for the PBMC study. For SAT, 39 samples (*n* = 8, *n* = 10, *n* = 9, *n* = 12, respectively) were available. The mRNA expression was measured at baseline and 12 weeks by TaqMan® Low Density Array.

**Results:**

In PBMCs, LF decreased *ICAM1* mRNA expression (*P* < 0.05), which was different (*P* = 0.06, Bonferroni correction) from the observed increase in the FF group (*P* < 0.05). Also, compared to the control group, LF decreased *ICAM1* mRNA expression (*P* < 0.05). Moreover, the change in *ICAM1* mRNA expression correlated positively with the intake of FF (*P* < 0.05) and negatively with the intake of LF (*P* < 0.05), independently of study group. A diet enriched in CSO, a rich source of alpha-linolenic acid (ALA), decreased PBMC *IFNG* mRNA expression (*P* < 0.01). The intake of CSO in the CSO group, but not the increase in plasma ALA proportions, correlated inversely with the *IFNG* mRNA expression in PBMCs (*P* = 0.08). In SAT, when compared with the control group, the effect of FF on decreasing *IL1RN* mRNA expression was significant (*P* < 0.03).

**Conclusion:**

We propose that CSO intake may partly exert its benefits through immuno-inflammatory molecular regulation in PBMCs, while modulation of *ICAM1* expression, an endothelial/vascular-related gene, may be more dependent on the type of fish consumed.

## Introduction

Dietary sources of *n*-3 fatty acids (FAs), e.g., fish as a source of eicosapentaenoic acid (EPA, 20:5 *n*-3) and docosahexaenoic (DHA, 22:6 *n*-3) and vegetable oils rich in alpha-linolenic acid (ALA, 18:3 *n*-3), are regarded to have beneficial effects on serum lipid profile and glucose metabolism, even though still controversial^1–4^, potentially contributing to a protective effect against type 2 diabetes (T2D) and cardiovascular disease (CVD). Still, intake of lean fish (LF), a poor source of *n*-3 FAs as compared to fatty fish (FF), may reduce the risk of stroke and CV risk factors, including circulating markers related to inflammation^5–9^.

One of the possible mechanisms linking the protective effect of *n*-3 FAs could be through decreasing inflammation. Indeed, previous results have shown that different sources of *n*-3 FAs, mainly EPA and DHA, may affect gene expression levels of markers involved in inflammation and immune system^10–12^. The possible molecular mechanisms linking the protective effect of LF, on the other hand, have been much less studied. Even though we and others have found conflicting results in the effect of *n*-3 FAs or LF on the expression of immune-inflammatory related genes, these studies were performed in different populations and lacked an intervention arm with dietary *n*-3 FA content as vegetable oil or fat^1,13,14^.

Peripheral blood mononuclear cells (PBMCs) are readily accessible circulating cells that include lymphocytes and monocytes and play a central role in inflammation^15^, and likely in the development of CVD^16^ and T2D^17^. PBMC gene expression is suggested as a model to investigate the effect of dietary interventions on inflammation^15^. Low-grade inflammation has been proposed as an important link between obesity and its secondary consequences such as disturbances in lipid and glucose homeostasis resulting in CVD and T2D^18,19^. Adipose tissue cells, in turn, seem to respond to dietary modifications^20–24^. However, from both tissues, randomized control studies in humans are still scarce regarding the specific effect of different sources of *n*-3 FAs in subjects at high risk for developing CVD and T2D.

We have recently shown that a camelina sativa oil (CSO)-enriched diet improved serum lipid profile as compared with a diet enriched either in FF or LF in subjects with impaired fasting glucose^25^. In the present study, our primary aim was to examine in a randomized controlled setting whether FF, LF and ALA-rich CSO differ in their effects on the transcriptional response of selected genes related to inflammation in PBMCs. Because we had subcutaneous adipose tissue (SAT) from a subset out of these samples, we also evaluated the response of these inflammatory genes and the adiponectin gene (*ADIPOQ*) in SAT.

## Materials and methods

### Study population

Altogether, 96 Caucasian volunteers aged 40 to 75 years were recruited in Kuopio area, Finland, via advertisements in newspapers, noticeboards and intranet of the university, and from previous clinical trials at our Department^25^. The study inclusion criteria were: a fasting plasma glucose concentration 5.6–6.9 mmol/l, concomitant with a 2 h glucose concentration in the oral glucose tolerance test of <11.0 mmol/l, body mass index (BMI) 25–36 kg/m^2^, fasting serum total cholesterol <7 mmol/l, low-density lipoprotein-cholesterol (LDL-C) <5.0 mmol/l and total triglycerides <4.0 mmol/l. Subjects were excluded if they had: any chronic disease, a condition hampering the ability to follow the dietary intervention protocol, alcohol abuse (>40 g/d) and weight loss of >5% during the preceding 6 months.

Originally, 79 subjects who were randomized to one of the following 12-week intervention groups, FF (*n* = 20), LF (*n* = 21), CSO (*n* = 18) or a control group (*n* = 20), finished the trial^25^ (SFigure1). For details on drop-out rates and sample size calculations, see Supplementary Information (SI) and ref. ^25^.

For the PBMC gene expression study, we analyzed data from 72 participants (FF: *n* = 19; LF: *n* = 19; CSO: *n* = 17 and control group: *n* = 17) (Table 1). As described in SFigure 1, sample measurements from seven participants were lost for technical reasons during the gene expression procedure. To study SAT gene expression, we analyzed data from 39 participants (FF: *n* = 8, LF: *n* = 10; CSO: *n* = 9 and control group: *n* = 12) (STable 1) out of the 44 who originally volunteered for AT biopsy collection. One participant had SAT sample taken only at baseline, and the measurements from the other four participants were lost due to similar reasons as for the PBMCs (SFigure1).

**Table 1:** Baseline characteristics of the subjects participating in the peripheral blood mononuclear cells mRNA expression study (*n* = 72)

	Fatty fish (*n* = 19)	Lean fish (*n* = 19)	CSO (*n* = 17)	Control (*n* = 17)
Age, years	58.5 ± 6.7	57.3 ± 7.8	57.8 ± 5.6	60.2 ± 6.7
Sex, M/F (*n*)	9 / 10	10 / 10	8 / 9	8 / 9
Body weight, kg	83.0 ± 12.1	84.8 ± 14.0	83.3 ± 9.6	85.6 ± 9.3
BMI, kg/m^2^	29.3 ± 2.2	29.7 ± 3.1	29.0 ± 2.2	29.4 ± 2.8
FPG, mmol/l	5.9 ± 0.4	6.1 ± 0.4	6.1 ± 0.4	6.0 ± 0.5
Serum cholesterol, mmol/l				
Total	5.1 ± 1.1	5.3 ± 1.1	5.3 ± 1.0	5.1 ± 0.9
HDL	1.4 ± 0.4	1.4 ± 0.4	1.3 ± 0.3	1.3 ± 0.3
LDL	3.0 ± 1.0	3.3 ± 0.9	3.2 ± 1.0	3.1 ± 0.8
Serum triglycerides, mmol/l	1.6 ± 0.8	1.3 ± 0.6	1.6 ± 0.6	1.5 ± 0.6
Use of statins, *n*	5	4	4	5
Serum fasting hsCRP, mg/l	2.23 ± 1.25	1.91 ± 1.45	2.3 ± 2.5	2.15 ± 2.00

Data are mean ± SD or *n*

*FPG* fasting plasma glucose, *hsCRP* high-sensitive C-reactive protein

### Original study design and interventions

Recruitment for AlfaFish study started in autumn 2012 and it was completed in June 2014. After a 4-week run-in phase in which the subjects followed their conventional diet and were not allowed to use any oil supplements or products enriched in plant stanols or sterols, the subjects were randomly assigned into a CSO, LF, FF or control group for 12 weeks. Randomization was stratified by sex, age and use of statins. The subjects visited the study clinic at 0 (baseline), 4, 8 and 12 weeks (end of study). Physical activity, alcohol intake, smoking, body weight and use of medication were to be kept constant during the study.

The study diets were isocaloric including current nutrient recommendations^26^, excluding fish and ALA intakes. Subjects in the FF and LF groups were instructed to consume four meals of fish per week (100–150 g per meal). For example, in the FF group, salmon, rainbow trout, Baltic herring, vendace, whitefish and mackerel to provide around 1 g of EPA + DHA per day, and in the LF group, for example, pike, perch, pike-perch, saithe and cod. Both fish groups were asked to decrease the intake of meat. The CSO group ingested CSO (27 g) in order to get 10 g of ALA per day and participants were asked to decrease the intake of other vegetable oils. The control and CSO groups were allowed to eat one fish meal per week and were instructed to replace some of the fish meals by lean meat and skin-free chicken. In the control group, the subjects were advised to keep the intake of dietary fats unchanged.

FA composition of plasma phospholipids and cholesteryl esters (CE) was determined by gas chromatography^27^, with an exception of using C19:0 as an internal standard instead of C17:0, to assess compliance with the study diets. Moreover, food records and daily consumption records of key food items in both fish groups and CSO group were kept for monitoring compliance.

The study was approved by the Ethical committee of the Hospital District of Northern Savo (55/2012). The subjects gave written informed consent after receiving both oral and written information.

### Collection of the samples

At both baseline (0 week) and end of the study (12 weeks) visits, blood samples were drawn after a 10-h overnight fasting from an antecubital vein for the biochemical parameters, including primary end points previously reported^25^, and PBMCs. The PBMCs were isolated within 45 to 60 min from the blood samples collected using special tubes developed for this purpose (cell preparation tubes: CPT) according to the manufacturer’s instructions (Becton, Dickinson and Company, Franklin Lakes, NJ, USA). Separated PBMCs were suspended in lysis buffer and stored at −80 °C until RNA extraction^13^. In a separate visit within the same week, SAT biopsy specimen was obtained by needle aspiration just below and lateral to the umbilicus under local anesthesia (1% lidocaine without adrenalin)^23,28^. Right after the biopsy, samples were washed twice to remove blood contamination, subsequently frozen in liquid nitrogen and stored at −80 °C until RNA extraction.

### Isolation and extraction of RNA

The RNA from PBMC and SAT samples was extracted using the miRNeasy Mini Kit (Cat. No. 217004, Quiagen GmBH, Hilden, Germany) in accordance with the manufacturer’s protocol and stored in RNase-free water at −75 °C.

### cDNA synthesis and real-time qPCR

The following work was performed at the Karolinska Institute (Sweden). The RNA integrity was checked using a Bioanalyzer device (Agilent 2100 Bioanalyzer, Agilent Technologies, Santa Clara, CA, USA). Then, 200 ng RNA was converted to complementary DNA (cDNA) using First-Strand Synthesis SuperMix (p/n 11752–050) from ThermoFisher Scientific (Waltham, MA, USA).

The expression levels of selected target genes and internal control genes (*GAPDH* and *TBP* for PBMCs; *18S* and *LRP10* for SAT) were measured on 384-well TaqMan® Low Density Arrays (ThermoFisher Scientific). Then, 100 ng (1 ng/μl) of cDNA was dispersed on the arrays together with TaqMan® gene expression master mix (Cat. No. 4369016, ThermoFisher Scientific). Amplification was registered in a 7900HT Fast Real-Time PCR System (ThermoFisher Scientific). All samples were run in duplicate. The resulting data were analyzed with RQ Manager 1.2.1 (Life Technologies). Data Assist software 3.01 (ThermoFisher Scientific) was used to normalize results in relation to expression of the internal control genes *GAPDH* and *TBP* (for PBMCs), and *18S* and *LRP10* (for SAT). Ct values from one sample was used as a reference in the calculations of relative gene expression (the delta delta Ct method) for normalization of all other samples.

For evaluating inflammation-related molecules at the messenger RNA (mRNA) expression levels, we selected *IL1B, IL1RN, IL6, IL10, TNF, TNFRSF1A, TNFRSF1B, TLR2, TLR4, RELA, ICAM1* and *CCL2* in addition to *IL18* and *IFNG* for PBMCs and *ADIPOQ* for SAT for data analyses. The target genes were selected based on our previous dietary studies in PBMCs and AT investigating inflammation at the transcriptomic level^13,15,21^ and on studies potentially showing an effect of different sources of PUFAs on immune-inflammation-related genes in PBMCs^29,30^.

### Statistical analyses

Analyses of dietary, biochemical, clinical and mRNA gene expression variables were performed using SPSS version 23.0 (IBM Corp., Armonk, NY.). The normality of the variables was tested with the Kolmogorov–Smirnov test followed by histogram plotting. When appropriate, skewed variables were log-10-transformed before analyses.

To compare the effect of each of the intervention groups on the fold change of the gene mRNA expression in PBMCs and SAT, and in plasma FA composition (12-week value minus 0-week value) we used analysis of covariance (ANCOVA) models. ANCOVA included each of the outcome of interest as the dependent variable and its baseline value as covariate. Quade’s test was used when any of the variables included in the analysis were not valid for parametric tests even after log-10 transformation. Either ANCOVA or Quade’s test models included the study group as the fixed effect, in which each of the outcome of interest was the dependent variable and its baseline value was covariate. The control group was used as a reference group when comparing group differences. Whenever a significant group effect was observed, Bonferroni correction for multiple testing was applied, and within-group changes (0 vs. 12 weeks) were tested by paired sample *t*-test (for variables or log-10 transformed variables that achieved normal distribution) or Wilcoxon signed ranks test (for non-parametric variables).

As secondary analyses, we also tested within-group changes whenever the effect of one of the study group vs. control group (represented by the β-coefficient) in the above-described models testing gene expression response had a *P*-value < 0.10 (e.g., *IFNG* in PBMCs and *IL1RN* in SAT). Spearman's correlations (rho (*ρ*)) were used to relate relevant changes in the mRNA expression levels with the changes in plasma FA composition of CE and phospholipid fractions and with the intakes of fatty or lean fish and CSO. The data are reported as mean ± SD for normally distributed variables or median (interquartile range (IQR)) for variables with non-normal distribution. A *P* value < 0.05 was considered to be statistically significant.

## Results

### Dietary intake and plasma *n*-3 FA composition related to compliance

Participants kept their body weight unchanged during the study (time effect, *P* = 0.31; group vs. time effect, *P* = 0.35). The nutrient intake and dietary compliance assessed by plasma FA composition in all study subjects has been reported in detail elsewhere^25^. Similarly, subjects participating in the PBMC gene expression study reported higher intake of ALA during the intervention in the CSO group as compared with the other groups (*P* < 0.05). The intakes of EPA and DHA were higher in the FF group than in the LF and control groups (*P* < 0.05 and *P* < 0.07, respectively; Table 2). Furthermore, the average numbers of fish meals per week during the study were 4.4 ± 0.4, 4.3 ± 0.5, 0.9 ± 0.4 and 0.9 ± 0.5 in the FF, LF, CSO and control groups, respectively. The consumption of CSO was 28.0 ± 2.7 g per day in the CSO group. These numbers were also similar to those previously reported.

**Table 2:** Dietary energy and fatty acid intake recorded by the subjects included in the peripheral blood mononuclear cells mRNA expression study (*n* = 72)

	Fatty fish, *n* = 19		Lean fish, *n* = 19		CSO, *n* = 17		Control, *n* = 17		
	0 Week	12 Weeks	0 Week	12 Weeks	0 Week	12 Weeks	0 Week	12 Weeks	*P* ^1^
Energy, kcal	1886 ± 562	2087 ± 512	2080 ± 473	2147 ± 437	2043 ± 529	2236 ± 624	1889 ± 427	1896 ± 444	0.28
Energy, kJ	7897 ± 2353	8729 ± 2144	8709 ± 1979	9104 ± 1836	8515 ± 2210	9361 ± 2616	7908 ± 1787	7939 ± 1859	0.12
Total fat, E%	35.7 ± 6.6	39.1 ± 5.2	34.2 ± 5.0	34.3 ± 3.3	35.3 ± 5.8	42.2 ± 3.2	33.8 ± 7.3	34.6 ± 5.0	0.001^2^
SFA, E%	12.1 ± 3.1	12.4 ± 2.5	11.7 ± 2.0	11.0 ± 1.8	12.3 ± 3.7	12.0 ± 1.9	11.6 ± 2.3	11.6 ± 2.0	0.53
MUFA, E%	13.0 ± 2.7	15.1 ± 2.1	12.1 ± 2.4	13.4 ± 1.9	12.4 ± 2.4	14.9 ± 1.6	11.8 ± 2.6	13.3 ± 2.6	0.62
PUFA, E%	6.1 ± 2.1	7.0 ± 1.4	5.8 ± 1.2	6.1 ± 0.8	6.1 ± 1.7	11.5 ± 1.6	5.9 ± 1.6	5.6 ± 1.0	<0.001^3^
LA, g	8.7 ± 4.5	10.9 ± 4.2	8.4 ± 3.6	11.5 ± 3.8	9.3 ± 3.9	13.5 ± 3.2	8.1 ± 3.4	8.9 ± 3.0	0.08
ALA, g	1.92 (1.28–2.73)	2.42 (1.93–3.33)^2^	1.85 (1.52–2.26)	2.65 (1.95–3.48)^2^	2.01 (1.47–2.87)	12.3 (11.8–13.12)	1.8 (1.37–1.8)	2.02 (1.65–2.87)	<0.001^3^
EPA, mg	70.4 (10.7–221)	436 (374–603)	105 (41.7–249)	38.6 (35.7–62.3)	118 (44.6–217)	106 (65.4–170)	34.4 (10.2–135)	57.1 (31.4–100)	<0.001^4^
DHA, mg	173 (55.4–306)	1090 (795–1300)	218 (99.7–645)	134 (94.1–168)	290 (118–578)	301 (142–395)	120 (45.7–352)	163 (85.8–306)	<0.001^5^

Dietary energy and fatty acid intake recorded by the subjects included 4-day food record at baseline, and mean of three 4-day food records during the intervention

Data are mean ± SD or median (IQR)

*ALA* alpha-linolenic acid, *CSO* camelina sativa oil, *DHA* docosahexaenoic acid, *EPA* eicosapentaenoic acid, *LA* linoleic acid, *MUFA* monounsaturated fatty acid, *PUFA* polyunsaturated fatty acid, *SFA* saturated fatty acid

^1^Study group vs. time, repeated measures general linear model

^2–5^Post hoc pairwise comparisons with Bonferroni correction:

^2^*P* < 0.05 for CSO group vs. lean fish and control groups

^3^*P* < 0.05 for CSO group vs. fatty fish, lean fish and control groups

^4^*P* < 0.05 for fatty fish group vs. control and lean fish group

^5^*P* < 0.05 for fatty fish group vs. control group

There was a significant increase in the proportion of ALA in plasma CEs and phospholipids in the CSO group (*P* < 0.001) as compared with the other groups. The proportion of EPA and DHA increased in the FF group in both lipid fractions as compared with the other groups (*P* < 0.01), except that in the post hoc tests DHA did not differ from the LF group in the phospholipid fraction (Table 3).

**Table 3:** The *n*-3 fatty acid composition in cholesteryl esters (CE) and phospholipid (PL) fractions at the baseline (0 week) and after 12-week interventions included in the peripheral blood mononuclear cell mRNA expression study (mean ± SD)

	Fatty fish, *n* = 19		Lean fish, *n* = 19		CSO, *n* = 17		Control, *n* = 17		
	0 Week	12 Weeks	0 Week	12 Weeks	0 Week	12 Weeks	0 Week	12 Weeks	*P* ^1^
Fatty acids in CE (mol%)									
18:3n-3 (α-linolenic acid)	1.04 ± 0.28	1.21 ± 0.48	1.20 ± 0.23	1.13 ± 0.27	1.08 ± 0.28	2.34 ± 0.59	1.00 ± 0.30	1.01 ± 0.25	<0.001^2^
20:5n-3 (EPA)	1.66 (1.4–2.04)	2.75(2.34–3.31)	2.18 (1.76–2.67)	1.94 (1.49–2.17)	2.18 (1.71–2.50)	2.61 (1.48–3.32)	2.01 (1.29–2.51)	1.69 (1.24–2.59)	<0.001^3^
22:6n-3 (DHA)	1.00 ± 0.30	1.16 ± 0.25	0.99 ± 0.23	0.99 ± 0.16	1.06 ± 0.22	0.93 ± 0.20	1.11 ± 0.17	1.06 ± 0.24	<0.001^4^
Fatty acids in PL (mol%)									
18:3n-3 (α-linolenic acid)	0.36 ± 0.12	0.41 ± 0.16	0.42 ± 0.10	0.42 ± 0.10	0.39 ± 0.12	0.79 ± 0.24	0.33 ± 0.10	0.34 ± 0.10	<0.001^2^
20:5n-3 (EPA)	1.56 (1.4–2.24)	2.67 (2.37–2.97)	1.97 (1.63–2.38)	1.81 (1.5–1.99)	1.84 (1.59–2.68)	2.67 (1.72–3.09)	1.83 (1.29–2.54)	1.83 (1.16–2.59)	<0.001^3^
22:6n-3 (DHA)	5.79 ± 1.33	6.44 ± 1.06	5.54 ± 1.16	5.43 ± 1.03	5.81 ± 1.34	5.24 ± 1.26	6.08 ± 0.86	5.72 ± 1.09	<0.001^5^

Data are mean ± SD or median (IQR)

*CSO* camelina sativa oil, *DHA* docosahexaenoic acid, *EPA* eicosapentaenoic acid, *PBMC* peripheral blood mononuclear cell

^1^For the effect of study group on fold changes adjusted for 0 week value, ANCOVA

^2–5^Post hoc pairwise comparisons with Bonferroni correction:

^2^*P* < 0.001 for CSO group vs. fatty fish, lean fish and control groups

^3^*P* < 0.001 for fatty fish group vs. lean fish and control groups, and *P* < 0.05 for fatty fish group vs. CSO group

^4^*P* < 0.05 for fatty fish group vs. lean fish and control groups, and *P* < 0.001 for fatty fish group vs. CSO group

^5^*P* < 0.01 for fatty fish group vs. CSO group, and *P* < 0^.^05 for fatty fish group vs. control group

The dietary FA intake and plasma FA composition of subjects taking part in the SAT gene expression study are described in STables 2 and 3. Overall, the compliance to the diet as assessed by these means and by the intake of CSO and fish during the study (data not shown) was similar to those earlier reported^25^.

### Effect of interventions on gene expression in PBMCs

**Fig. 1:**
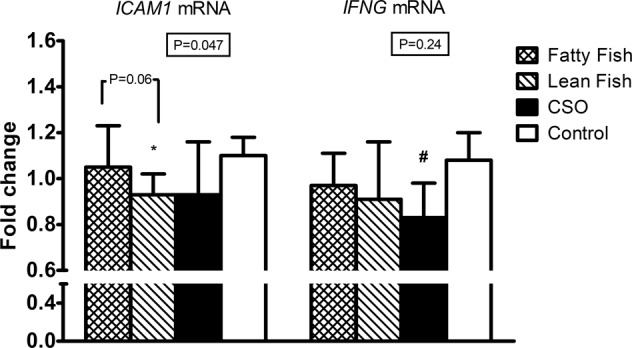
Fold changes (12–0 weeks) in *ICAM1* and *INFG* mRNA expressions in peripheral blood mononuclear cells (12–0 weeks) in each of the study groups. Group effect between fold changes adjusted for mRNA expression at 0 week are indicated inside the boxes for each marker and were tested by Quades’s test for *ICAM1* or ANCOVA for *IFNG*, both followed by Bonferroni’s post hoc tests for multiple comparisons. For the effect of the study group vs. control in the respective model: *P = 0.025 and ^#^*P* = 0.087. CSO camelina sativa oil

Data on mRNA expression levels from the PBMC samples before and after the dietary interventions are depicted in Table 4 and their respective fold changes in STable 4. There were no significant differences among the groups in the mRNA expression change of most of the markers, except for *ICAM1* and *IFNG* (Fig.1). The change in *ICAM1* mRNA expression in the LF group (median (IQR): 0.93 (0.75–1.02)) tended to be significantly different from the change observed in the FF group (1.05 (0.99–1.23), *P* = 0.06; Fig.1). While *ICAM1* mRNA expression decreased in the LF group, its expression increased in the FF group (*P* < 0.05 for both, Table 4). Nevertheless, as compared to the control group, only LF had a significant effect on *ICAM1* mRNA levels (β-coefficient ± SD: −15.3 ± 6.7, *P* = 0.025), but not FF or CSO groups (*P* > 0.10 for both FF and CSO in the model).

**Table 4:** Relative gene mRNA expression of immune-inflammatory-related molecules from peripheral blood mononuclear cells at baseline (0 week) and end of the study (12 weeks) in each of the study groups (*n* = 72)

	Fatty fish, *n* = 19		Lean fish, *n* = 19		Camelina sativa Oil, *n* = 17		Control, *n* = 17		
	0 Week	12 Weeks	0 Week	12 Weeks	0 Week	12 Weeks	0 Week	12 Weeks	*P* ^1^
*CCL2*	2.14 (1.30–4.50)	2.10 (1.08–3.24)	3.08 (1.00–4.23)	1.74 (1.12–3.83)	2.33 (1.40–3.50)	2.50 (1.12–3.76)	3.08 (1.00–4.39)	1.82 (0.93–3.77)	0.98
*ICAM1*	1.66 (1.32–2.39)	2.03^2^ (1.24–2.75)	1.73 (1.47–2.53)	1.50^2^ (1.15–2.49)	2.17 (1.40–2.93)	2.06 (1.55–2.60)	1.64 (1.15–3.00)	1.87 (1.36–2.50)	0.047^3^
*IL1RN*	1.95 (1.36–3.34)	2.45 (1.38–3.85)	2.23 (1.46–3.84)	2.10 (1.46–4.57)	3.06 (1.54–4.48)	2.83 (1.60–3.71)	1.82 (1.17–4.42)	2.30 (1.55–4.14)	0.92^3^
*IL1B*	1.06 (0.70–1.64)	1.22 (0.74–1.41)	1.18 (0.85–2.11)	1.15 (0.80–1.85)	1.10 (0.85–1.69)	1.25 (0.87–1.59)	1.07 (0.59–2.25)	1.17 (0.82–2.00)	0.75
*IL6*	1.23 (0.80–2.37)	1.08 (0.66–2.49)	1.00 (0.56–1.78)	0.77 (0.47–2.45)	2.45 (0.85–5.25)	1.41 (0.93–3.64)	0.94 (0.43–1.64)	1.08 (0.73–1.97)	0.78
*IL10*	3.92 (2.64 –26.4)	5.21 (1.91–22.5)	10.2 (3.50–24.2)	6.62 (2.84–18.9)	5.95 (2.98–28.4)	8.57 (2.93–22.6)	5.10 (2.77–27.1)	5.17 (1.96–26.6)	0.96
*IL18*	2.02 (1.27–3.14)	1.73 (1.17–2.98)	2.38 (1.18–3.63)	2.16 (1.16–4.11)	2.39 (1.23–3.70)	2.14 (1.48–3.23)	1.58 (1.12–4.11)	2.03 (1.29–3.82)	0.54
*TNF*	1.05 (0.87–1.29)	1.17 (0.89–1.56)	1.22 (0.92–1.65)	1.22 (0.77–1.78)	1.11 (0.75–1.63)	1.07 (0.80–1.59)	1.03 (0.76–1.60)	1.16 (0.70–1.59)	0.88
*TNFRSF1A*	1.54 (1.05–2.45)	1.67 (0.92–2.68)	1.31 (1.01–2.26)	1.24 (0.93–2.51)	1.73 (0.98–2.54)	1.68 (1.10–2.41)	1.34 (1.01–2.13)	1.46 (1.09–2.16)	0.87^3^
*TNFRSF1B*	1.20 (0.85–1.62)	1.33 (0.88–2.00)	1.34 (1.00–1.94)	1.09 (0.91–1.86)	1.48 (0.89–1.87)	1.50 (1.08–1.84)	1.05 (0.83–2.01)	1.21 (0.91–1.70)	0.24^3^
*RELA*	0.87 (0.70–1.90)	0.95 (0.73–1.76)	1.39 (0.84–1.96)	0.97 (0.78–1.79)	1.88 (0.90–2.10)	1.61 (0.91–1.94)	0.87 (0.79–2.03)	1.09 (0.77–1.69)	0.27
*TLR2*	2.73 (1.81–4.08)	2.27 (2.00–3.83)	3.22 (1.66–4.44)	2.40 (1.83–3.45)	2.58 (1.76–4.25)	3.29 (2.09–4.89)	2.08 (1.80–4.46)	2.39 (2.02–3.88)	0.09
*TLR4*	2.04 (1.36–3.07)	1.90 (1.29–3.38)	2.49 (1.41–3.76)	1.98 (1.43–2.94)	2.48 (1.52–3.26)	2.15 (1.64–3.35)	1.68 (1.41–3.55)	1.95 (1.64–3.45)	0.21^3^
*IFNG*	0.82 (0.49–1.35)	0.83 (0.42–1.43)	1.00 (0.61–1.67)	0.90 (0.49–2.05)	1.36 (0.90–1.67)	1.09^4^ (0.60–1.20)	0.96 (0.64–1.38)	0.93 (0.61–1.34)	0.24

Data are median (IQR)

For the effect of study group on fold changes adjusted for mRNA expression at 0 week (^1^ANCOVA, ^3^Quade’s test)

^2^*P* < 0.05, Wilcoxon signed rank test for related samples

^4^*P* < 0.01, paired *t-*test for related samples

Although the overall difference among the groups did not reach significance for a distinct change in PBMC *IFNG* mRNA expression (*P* = 0.24, Table 4), there was a trend for PBMC *IFNG* mRNA expression to decrease in the CSO group (β-coefficient ± SD: −0.17 ± 0.1, *P* = 0.087, Fig. 1), but not in the other study groups (*P* > 0.50 for both FF and LF) in relation to the control group. In line with that, after applying the pairwise comparison within the CSO group, we indeed observed a significant decrease in the mRNA expression levels of *IFNG* (*P* < 0.01, Table 4).

### Effect of interventions on gene expression in SAT

Data on mRNA expression levels from SAT samples before and after the dietary interventions are depicted in Table 5 and their respective fold changes in STable 5. Although the overall group effect was not significant for any of the markers studied in SAT when compared to the control group, only the effect of FF on decreasing *IL1RN* mRNA expression in SAT was significant (β-coefficient ± SD: −0.41 ± 0.18, *P* = 0.03; STable 5), but not in the other study groups (*P* > 0.15 for both LF and CSO) in relation to the control group. In within-group comparisons, *IL1RN* expression levels tended to decrease in the FF group (*P* = 0.05, Table 5).

**Table 5:** Relative gene mRNA expression of immune-inflammatory-related genes from subcutaneous adipose tissue at baseline (0 week) and end of the study (12 weeks) in each of the study groups (*n* = 39)

	Fatty fish, *n* = 8		Lean fish, *n* = 10		Camelina sativa oil, *n* = 9		Control, *n* = 12		
	0 Weeks	12 Weeks	0 Week	12 Weeks	0 Week	12 Weeks	0 Week	12 Weeks	*P* ^1^
*CCL2*	2.12 (1.38–2.46)	1.72 (0.92–2.27)	1.60 (1.12–2.28)	1.90 (1.15–2.47)	1.60 (0.83–2.01)	1.27 (0.96–2.11)	1.62 (1.07–1.85)	1.52 (1.15–1.93)	0.67
*ICAM1*	1.68 (1.33–1.95)	1.78 (1.41–2.05)	1.80 (1.35–2.07)	1.72 (1.30–2.06)	1.47 (1.21–1.71)	1.50 (1.28–1.89)	1.72 (1.34–1.83)	1.76 (1.54–2.13)	0.97^2^
*IL1RN*	0.80 (0.58–1.49)	0.37^3^ (0.16–0.83)	0.44 (0.13–0.64)	0.37 (0.19–0.57)	0.58 (0.18–1.35)	0.33 (0.18–0.82)	0.47 (0.35–0.59)	0.58 (0.36–1.24)	0.12
*IL1B*	3.78 (2.38–7.74)	3.19 (0.91–8.53)	3.26 (1.80–10.26)	4.67 (1.91–6.57)	3.02 (2.11–3.87)	1.51 (1.09–3.12)	3.43 (1.33–6.07)	3.21 (2.46–4.25)	0.60
*IL6*	1.09 (0.92–1.51)	1.48 (0.44–2.26)	1.03 (0.58–1.48)	1.19 (0.97–1.55)	1.15 (0.70–1.98)	1.32 (0.72–2.17)	0.91 (0.71–1.38)	0.66 (0.56–1.27)	0.74^2^
*IL10*	2.61 (1.13–3.20)	2.11 (0.66–2.66)	1.46 (1.10–2.01)	1.71 (1.13–2.99)	1.32 (1.16–1.71)	1.31 (1.04–2.32)	1.72 (1.01–1.98)	1.27 (0.98–2.88)	0.63
*TNF*	2.71 (2.49–3.64)	3.07 (2.05–4.48)	3.23 (2.26–5.44)	3.33 (1.99–4.75)	1.81 (1.57–3.69)	1.85 (1.34–2.85)	2.96 (1.91–3.79)	3.33 (2.78–3.56)	0.31
*TNFRSF1A*	3.15 (1.35–4.85)	3.41 (1.74–4.77)	4.40 (2.62–5.41)	4.08 (2.27–5.99)	3.87 (3.04–5.13)	3.66 (3.32–4.43)	4.30 (2.72–5.49)	3.80 (3.09–5.03)	0.97^2^
*TNFRSF1B*	1.50 (1.18–1.70)	1.38 (1.22–1.95)	1.61 (1.18–1.69)	1.46 (1.19–1.61)	1.13 (0.92–1.32)	0.92 (0.82–1.14)	1.43 (0.99–1.75)	1.33 (1.18–1.62)	0.59
*RELA*	1.16 ± 0.23	1.31 ± 0.29	1.18 ± 0.25	1.18 ± 0.17	1.22 ± 0.27	1.13 ± 0.21	1.33 ± 0.17	1.21 ± 0.19	0.20
*TLR2*	2.26 (1.30–4.58)	1.93 (1.48–7.20)	2.71 (2.27–4.28)	3.24 (2.17–3.48)	2.35 (1.81–2.69)	2.05 (1.64–2.74)	2.11 (1.45–3.56)	2.33 (1.81–2.99)	0.94^2^
*TLR4*	1.50 (1.39–1.71)	1.69 (1.46–2.07)	1.59 (1.17–1.83)	1.64 (1.56–1.91)	1.59 (1.30–1.86)	1.50 (1.40–1.69)	1.59 (1.32–2.06)	1.66 (1.36–2.01)	0.34
*ADIPOQ*	1.73 (1.28–2.13)	1.89 (1.73–2.20)	1.66 (1.32–2.26)	1.57 (1.41–2.07)	1.89 (1.49–2.18)	1.67 (1.41–2.09)	1.91 (1.52–2.24)	1.68 (1.48–2.28)	0.19

Data are mean ± SD or median (IQR)

For the effect of study group on fold changes adjusted for mRNA expression at 0 week (^1^ANCOVA, ^2^Quade’s test)

^3^*P* = 0.05, Wilcoxon signed rank test for related samples

### Correlations of changes in the mRNA expression of genes modulated by the diets with the changes in plasma FAs and the reported fish and CSO intakes during the study

The change in *ICAM1* mRNA expression in PBMCs was positively correlated with the intake of fatty fish (portions per week) (*ρ* = 0.24, *P* = 0.046), and negatively correlated with the intake of lean fish (portion per week) (*ρ* = −0.27, *P* = 0.025). The associations between changes in I*CAM1* mRNA expression with FF and LF intakes were only observed when we considered the entire study population in the analyses, but not when tested within each of the relevant study groups (FF group: *ρ* = −0.02, *P* = 0.94 and *ρ* = −0.39, *P* = 0.10, respectively; LF group: *ρ* = 0.12, *P* = 0.64 and *ρ* = 0.11, *P* = 0.65, respectively).

However, in general we did not find any consistent correlation between the changes in mRNA expression levels of *ICAM1* and in *n*-3 FAs in both CE and phospholipid fractions. None of these correlations were significant when considering the whole study population (*P* > 0.10, STable 6). The same was observed within the FF group (*n* = 19, *P* > 0.30; STable 6), except for a trend for an inverse correlation between the increases in *ICAM1* mRNA expression and DHA proportions in CEs (DHA: *ρ* = −0.40, *P* = 0.088).

The changes in PBMC *IFNG* mRNA expression correlated with the reported intake of CSO (portions per day) (*n* = 17; *ρ* = −0.44, *P* = 0.08), but did not correlate with the changes in ALA proportions in either CE or PL fractions (*n* = 72; *ρ* = −0.17, *P* = 0.15 and *ρ* = −0.16, *P* = 0.19, respectively; CSO group, *n* = 17: *ρ* = −0.06, *P* = 0.82 and *ρ* = −0.23, *P* = 0.38, respectively). Because of the previously reported beneficial effect of CSO on serum lipid profile, we also tested the correlation between the changes in *IFNG* expression and plasma lipids. We observed that, when considering all the subjects (*n* = 72), the change in *IFNG* mRNA expression was positively correlated with the changes in total cholesterol (TC: *ρ* = 0.40, *P* = 0.001), LDL-C (*ρ* = 0.35, *P* = 0.001), TC to high-density lipoprotein-cholesterol (HDL-C) ratio (*ρ* = 0.37, *P* = 0.001) and ApoB to ApoA-I ratio (*ρ* = 0.32, *P* = 0.006).

Finally, the change of *IL1RN* mRNA expression in SAT after the study correlated negatively with the change in EPA in the plasma CE fraction (*ρ* = −0.32, *P* = 0.044). However, we did not find any other further correlations with serum *n*-3 FA proportions (STable 6).

## Discussion

In this study, we investigated the effects of fish and CSO intakes on the changes in gene expression in PBMCs and SAT samples from subjects at high risk of developing T2D and consequently CVD. Our results show that, even though modest, the PBMCs were more responsive to the dietary modifications than the SAT, which might have been in part due to the smaller sample size and lack of power in the analyses of SAT samples. In PBMCs, while a diet enriched in lean fish decreased the levels of *ICAM1* mRNA expression, a diet enriched in CSO, a rich source of ALA, decreased the levels of *IFNG* mRNA expression.

The mRNA expression of *ICAM1* was downregulated after LF in PBMCs. High consumption of fish, especially of lean fish, may reduce risk of stroke^7^ and cardiovascular lipid risk factors in healthy subjects^8^, and blood pressure levels in subjects with coronary heart disease^5^. Moreover, LF contains a number of nutrients that may be beneficial in the prevention of CVD^31^. One example is taurine, an amino sulfonic acid, present in fish and in higher content in LF^31–33^, which has been related to improved vascular endothelial function^34^, also at the molecular level^35,36^. *ICAM1* encodes for an inflammation-related protein named intercellular adhesion molecule 1 (ICAM-1) that is known as a marker of endothelial dysfunction^37,38^, a key event in CVD. Even though we did not see any changes in serum ICAM-1 circulating levels after 12 weeks of a LF diet or a different effect among the study groups^25^, we can observe in this population study a positive correlation between *ICAM1* mRNA expression in PBMCs and ICAM-1 circulating levels in serum at both baseline and 12 weeks (data not shown). Furthermore, it is also possible that the effect of LF on serum ICAM-1 circulating levels is more evident in patients with coronary heart than in individuals still free of CVD.

We cannot explain the reason why in the FF group we observed an increase in the expression of *ICAM1*. Rundblad et al.^1^ observed that an 8-week fish diet consisting of lean fish and fatty fish decreased circulating levels of ICAM1 compared to baseline. We also observed a negative correlation between the intake of FF during the study and the changes in *ICAM1* mRNA expression. Other factors or nutrients than the FAs may explain our results. For example, FF depending from where it comes can contain high amounts of mercury, which might impair the protective effects of fish and omega-3 FAs and increase inflammation^39^. However, this is unlikely to play a major role, because most of the FF consumed by the participants were farmed fish, which has low content of mercury^40^.

An interesting finding was the correlation between the increase in CSO, but not in ALA plasma proportions, with the decrease in the PBMC *IFNG* mRNA expression levels in the CSO group. *IFNG* encodes cytokine interferon-gamma (IFN-γ), which has important immunoregulatory functions and is a potent activator of macrophages^41^. The encoded protein is secreted by cells of both the innate and adaptive immune systems. A higher ALA intake provided as flaxseed oil was shown to decrease INF-γ mRNA expression and proliferation of T cells in a dose-dependent manner in mice^42^, but did not seem to influence immune function in humans^29,30^. Still, our findings could reflect immune response since the referred studies used ALA intakes that varied from 2 to 3.5 g/day from flaxseed oil, while we provided an average of 10 g/day from CSO. These results are important due to the role of the immune system in metabolic health and diseases such as T2D^43^. Along with its benefit on serum lipid profile earlier observed^25^, CSO could be possibly promoting CV health in long-term through molecular mechanisms related to immunoregulatory functions^44^. In fact, changes in PBMC *INFG* expression correlated with the changes in lipid profile induced by CSO.

Regarding the SAT mRNA expression response to the dietary interventions, we observed a slight decrease in *IL1RN* expression in the FF group of ≈5%. Recent work has confirmed the limited effect of fish oil supplementation on SAT gene expression at least in healthy individuals^45^. In this same work, though, *IL1RN* was among the regulated genes that benefited from fish oil supplementation during evoked adipose tissue inflammation^45^. SAT is an important source of interleukin-1 (IL-1) family cytokines, including IL-1 receptor antagonist (IL-1Ra)^46^. Elevated circulating levels of IL-1Ra in humans is associated with an increased risk to develop T2D^47^ with an accelerated increase just prior the disease onset^48,49^. Although we did not observe any changes in circulating levels of IL-1Ra in the original study population^25^, pairwise comparison within the FF group showed a reduction in this marker at borderline significance level (*P* = 0.07). Therefore, this immune-inflammatory response might be important, leading to the hypothesis that increasing the intake of FF could have a protective effect against development of T2D in the long term.

Among the inflammation-related markers studied in PBMCs, we were not able to find any other diet-induced response in mRNA expression other than the ones related to *ICAM1* and *IFNG*. An omega-3 index of >8% (EPA + DHA in red blood cells) is considered cardioprotective^50^. In the present study, the omega-3 index was relatively high at baseline, >8% for all groups^25^. Therefore, it is possible that a higher baseline content of these FAs in the common diet of the study participants have masked additional findings. Furthermore, previous studies with similar length investigating the response of immune-inflammatory genes in SAT have used much higher doses of EPA and DHA that are usually given as fish oil^51–53^. It is possible that lowering inflammation in SAT by increasing *n*-3 FAs would be more evident with concomitant weight loss or if studied in a more obese population^54^. Specifically related to *ADIPOQ*, EPA appears to regulate adiponectin levels at the translational or posttranslational level rather than at the transcriptional level^55^.

The strengths of the present study are the randomized controlled design and careful monitoring of the diet by both repeated food records, consumption records and relevant biomarkers. However, as power calculations were based on differences in DHA in plasma phospholipids, the small sample size of the present study, especially regarding the SAT sub-population, could have obscured broader and stronger findings at the mRNA expression levels. Moreover, the results are not generalizable to subjects with normal body weight and glucose metabolism. Furthermore, the significant within-group findings of *IFGN* in PBMCs and *IL1RN* in SAT should be taken cautiously due to lack of power to detect a group effect on these changes among the study groups in conventional statistical analyses.

In conclusion, we propose that CSO intake may exert its benefit through a molecular mechanism related to immunoregulatory function, since we observed decreased mRNA expression levels of IFN-γ after a 12-week diet enriched in CSO. Furthermore, an intake of lean fish four times per week may benefit cardiovascular health since we found reduced levels of *ICAM1* expression induced by a LF diet, when compared to a FF or a control diet.

## Supplementary information

Online Supplementary information
