# Supplementary material for: The effect of different sources of fish and camelina sativa oil on immune cell and adipose tissue mRNA expression in subjects with abnormal fasting glucose metabolism: a randomized controlled trial
Source: Nutr Diabetes. 2019 Jan 9;9:1. doi: 10.1038/s41387-018-0069-2 (PMC6347599; doi:10.1038/s41387-018-0069-2)
Supplement: Supplementary file 1 — Online Supplementary information [file 41387_2018_69_MOESM1_ESM.docx]

**Supplementary information on material, methods and results**

**1. Material and Methods**

**1.1. Study subjects randomized to the study**

Originally, from the 96 out of 153 recruited subjects who were eligible for the study, 79 who were randomized to one of the following 12-week intervention groups: fatty fish (FF) group (n=20), lean fish (LF) group (n=21), CSO group (n=18) or a control group (n=20) finished the trial (**SFigure 1**). The randomization was conducted by the study nurse based on a randomization table by matching the subjects according to sex, median of age, and use of statins. From every division of randomizing factors (gender→age→use of statins), it was possible to end up to any of the four groups. Before the randomization, eight subjects dropped out leaving 88 subjects to be randomized; 21 in the fatty fish and control groups, and 23 in the lean fish and ALA groups. Nine subjects dropped out within the first three weeks during the study. Altogether 79 subjects completed the intervention (SFigure 1).

The sample calculation was based on differences in DHA in serum phospholipids, a valid biomarker of dietary intake (n=18 per group, difference of 1.2 mol%, when alpha<0.05 and beta>0.9). This parameter was chosen due to modern methodology to be used in further analyses, e.g. gene expression, lipidomic and metabolomic profiles, for which it is not possible to select certain variables and determine clinically relevant changes.

**2. Results**

**Tables (6) and figure (1) in order of appearance in the main text**

**STable 1**. Baseline (0 week) characteristics of the participants who volunteered to collect adipose tissue (AT) biopsy included in the subcutaneous AT mRNA expression study (n=39)

|  | Fatty Fish,  n=8 | Lean Fish,  n=10 | CSO,  n=9 | Control,  n=12 | P^1^ |
| --- | --- | --- | --- | --- | --- |
| Age, years | 58.9 ± 7.2 | 60.3 ± 7.3 | 57.8 ± 5.9 | 61.3 ± 5.5 | 0.64 |
| Sex, male / female | 4 / 4 | 4 / 6 | 4 / 5 | 6 / 6 | 0.96 |
| BMI, kg/m^2^ | 28.8 ± 2.3 | 29.6 ± 2.1 | 29.2 ± 1.2 | 28.9 ± 2.4 | 0.78 |
| FPG, mmol/l | 6.1 ± 0.5 | 5.9 ± 0.5 | 5.8 ± 0.4 | 6.3 ± 0.8 | 0.38 |
| Serum cholesterol, mmol/l |  |  |  |  |  |
| Total | 5.3 ± 1.1 | 5.6 ± 1.1 | 5.2 ± 1.1 | 5.4 ± 1.1 | 0.84 |
| HDL | 1.3 ± 0.4 | 1.7 ± 0.5 | 1.2 ± 0.3 | 1.4 ± 0.3 | 0.05 |
| LDL | 3.3 ± 1.0 | 3.3 ± 0.7 | 3.1 ± 1.1 | 3.2 ± 1.1 | 0.96 |
| Serum triglycerides, mmol/l | 1.8 ± 1.0 | 1.1 ± 0.3 | 1.5 ± 0.7 | 1.6 ± 0.6 | 0.13 |
| Use of statins, *n* | 1 | 2 | 2 | 3 | 0.92 |
| Serum fasting hsCRP, mg/l | 2.14 ± 1.50 | 2.82 ± 3.47 | 2.73 ± 2.53 | 2.17 ± 2.01 | 0.81 |

CSO: camelina sativa oil. FPG: fasting plasma glucose. hsCRP: high sensitivity C reactive protein.

^1^ One-Way ANOVA (continuous variables) or χ^2^ test (categorical variables).


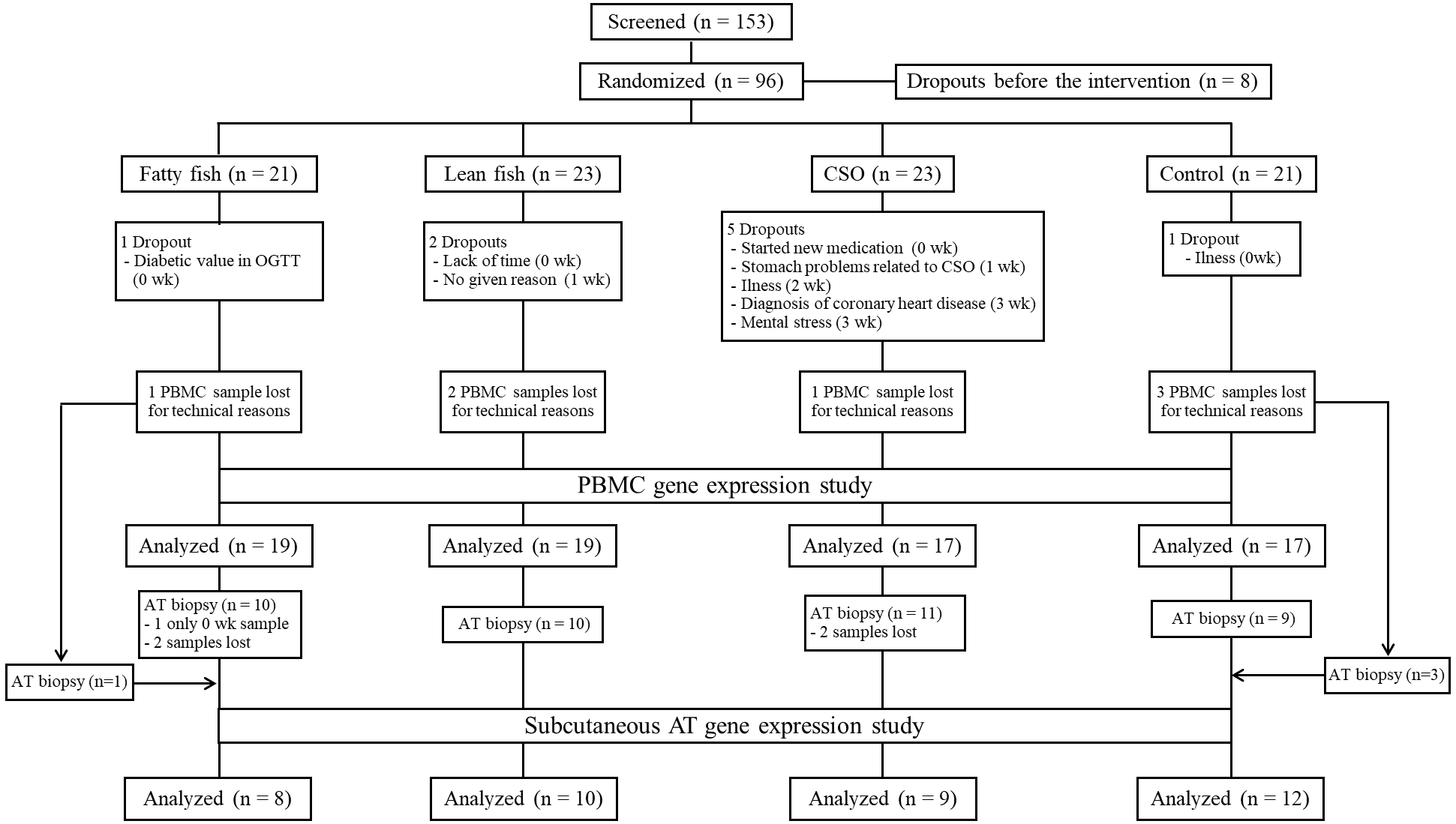


**SFigure 1**. Flowshart of the study. AT: adipose tissue, CSO: camelina sativa oil, OGTT: oral glucose tolerance test PBMC: peripheral blood mononuclear cells.

**STable 2.** Dietary energy and fatty acid intake recorded^1^ by the subjects included in the subcutaneous adipose tissue mRNA expression study (n = 39).

|  | Fatty fish, n = 8 | | Lean fish, n = 10 | | CSO, n = 9 | | Control, n=12 | |  |
| --- | --- | --- | --- | --- | --- | --- | --- | --- | --- |
|  | 0 week | 12 weeks | 0 week | 12 weeks | 0 week | 12 weeks | 0 week | 12 weeks | P^2^ |
| Energy, kcal | 2089 ± 489 | 2344 ± 639 | 1930 ± 618 | 2044 ± 476 | 2072 ± 479 | 2300 ± 498 | 1832 ± 185 | 1879 ± 392 | 0.60 |
| Energy, kJ | 8746 ± 2048 | 9812 ± 2676 | 8083 ± 2588 | 8559 ± 1996 | 8603 ± 1994 | 9632 ± 2084 | 7669 ± 776 | 7866 ± 1641 | 0.52 |
| Total fat, E % | 37.0 ± 4.4 | 38.7 ± 4.3 | 36.5 ± 5.9 | 34.3 ± 2.7 | 35.8 ± 6.2 | 41.8 ± 3.4 | 35.4 ± 7.4 | 35.2 ± 4.8 | 0.01 |
| SFA, E% | 14.0 ± 2.8 | 12.5 ± 2.5 | 11.7 ± 2.0 | 11.0 ± 1.8 | 12.3 ± 3.7 | 12.0 ± 1.9 | 11.6 ± 2.3 | 11.6 ± 2.0 | 0.54 |
| MUFA, E% | 12.4 ± 1.6 | 14.7 ± 1.7 | 12.1 ± 2.4 | 13.4 ± 1.9 | 12.4 ± 2.4 | 14.9 ± 1.6 | 11.8 ± 2.6 | 13.3 ± 2.6 | 0.28 |
| PUFA, E% | 5.5 ± 1.5 | 6.9 ± 0.8 | 5.8 ± 1.2 | 6.1 ± 0.8 | 6.1 ± 1.7 | 11.5 ± 1.6 | 5.9 ± 1.6 | 5.6 ± 1.0 | <0.001^3^ |
| ALA, g | 2.3 ± 0.8 | 3.1 ± 0.9 | 1.8 ± 0.9 | 2.7 ± 1.1 | 2.6 ± 0.9 | 13.0 ± 1.1 | 1.8 ± 0.57 | 2.03 ± 0.7 | <0.001^3^ |
| Linoleic acid, g | 8.7 ± 2.5 | 12.0 ± 2.6 | 7.5 ± 3.4 | 10.5 ± 3.1 | 10.7 ± 3.7 | 14.5 ± 2.7 | 7.8 ± 1.9 | 8.6 ± 2.4 | 0.15 |
| EPA, mg | 72 ± 68 | 606 ± 323 | 176 ± 184 | 122 ± 201 | 101 ± 87 | 99 ± 66 | 78 ± 88 | 91 ± 86 | 0.01^4^ |
| DHA, mg | 175 ± 183 | 1432 ± 920 | 458 ± 549 | 241 ± 269 | 270 ± 251 | 268 ± 180 | 237 ± 263 | 260 ± 242 | 0.003^5^ |

Data are mean ± SD. ALA, alpha-linolenic acid; CSO: camelina sativa oil; DHA, docosahexaenoic acid; EPA, eicosapentaenoic acid; MUFA: monounsaturated fatty acid; PUFA: polyunsaturated fatty acid; SFA: saturated fatty acid.

^1^ Four-day food record at baseline, mean of three 4-d food records during the intervention.

^2^ Study groups vs. time, repeated measures general linear model

^3-5^ Post-Hoc pairwise comparisons with Bonferroni correction:

^3^ P <0.001 for CSO group vs. fatty fish, lean fish and control groups.

^4^ P <0.05 for fatty fish group vs. control group.

^5^ P=0.07 for fatty fish group vs. control group.

**STable 3.** N-3 fatty acid composition in cholesteryl esters (CE) and phospholipid (PL) fractions at the baseline (0 week) and after 12 week interventions in subjects with SAT mRNA expression data (mean ± SD).

|  | Fatty fish, n=8 | | Lean fish, n=10 | | CSO, n=9 | | Control, n=12 | |  |
| --- | --- | --- | --- | --- | --- | --- | --- | --- | --- |
|  | 0 week | 12 weeks | 0 week | 12 weeks | 0 week | 12 weeks | 0 week | 12 weeks | P^1^ |
| *Fatty acids in CE (mol%)* |  |  |  |  |  |  |  |  |  |
| 18:3n-3 (α-linolenic acid) | 1.06 ± 0.24 | 1.16 ± 0.16 | 1.14 ± 0.38 | 1.13 ± 0.30 | 1.21 ± 0.22 | 2.44 ± 0.58 | 0.94 ± 0.26 | 0.97 ± 0.27 | <0.001^2^ |
| 20:5n-3 (EPA) | 1.95 ± 1.57 | 2.82 ± 1.14 | 2.19 ± 1.17 | 1.91 ± 0.39 | 2.36 ± 1.43 | 2.43 ± 1.01 | 2.18 ± 0.77 | 1.89 ± 0.75 | 0.005^3^ |
| 22:6n-3 (DHA) | 0.96 ± 0.35 | 1.13 ± 0.24 | 0.97 ± 0.29 | 0.97 ± 0.15 | 1.11 ± 0.25 | 0.90 ± 0.18 | 1.13 ± 0.24 | 1.07 ± 0.27 | 0.001^4^ |
| *Fatty acids in PL (mol%)* |  |  |  |  |  |  |  |  |  |
| 18:3n-3 (α-linolenic acid) | 0.38 ± 0.10 | 0.41 ± 0.09 | 0.41 ± 0.13 | 0.41 ± 0.12 | 0.44 ± 0.10 | 0.82 ± 0.23 | 0.29 ± 0.08 | 0.35 ± 0.11 | <0.001^2^ |
| 20:5n-3 (EPA) | 1.96 ± 1.64 | 2.64 ± 0.83 | 2.15 ± 1.29 | 1.75 ± 0.33 | 2.40 ± 1.57 | 2.79 ± 1.32 | 2.18 ± 0.84 | 2.12 ± 0.98 | 0.017^5^ |
| 22:6n-3 (DHA) | 5.57 ± 1.53 | 6.33 ± 0.82 | 5.25 ± 1.46 | 5.31 ± 1.01 | 5.85 ± 1.17 | 5.25 ± 1.24 | 6.32 ± 1.13 | 5.69 ± 1.18 | 0.013^4^ |

Data are mean ± SD. CSO: camelina sativa oil; DHA, docosahexaenoic acid; EPA, eicosapentaenoic acid; SAT: subcutaneous adipose tissue

^1^ For the effect of study group on fold changes adjusted for 0 week value, ANCOVA.

^2-5^ Post-Hoc pairwise comparisons with Bonferroni correction:

^2^ P <0.001 for CSO group vs. fatty fish, lean fish and control groups.

^3^ P <0.01 for fatty fish group vs. lean fish, and P <0.05 fatty fish group vs. control group.

^4^ P <0.01 for fatty fish group vs. CSO, and P=0.077 for fatty fish group vs. control group.

^5^ P <0.05 for fatty fish group vs. lean fish group.

**STable 4.** Changes^1^ in mRNA expression of inflammation-related genes from peripheral blood mononuclear cells in each of the study groups (n = 72).

|  | Fatty fish, n = 19 | Lean fish, n = 19 | Camelina Sativa Oil, n = 17 | Control, n = 17 |  |
| --- | --- | --- | --- | --- | --- |
|  | FC (median (IQR)) | FC (median (IQR)) | FC (median (IQR)) | FC (median (IQR)) | P^2^ |
| *CCL2* | 0.95 (0.80 – 1.19) | 0.86 (0.53 – 1.32) | 0.97 (0.68 – 1.40) | 1.12 (0.54 – 1.61) | 0.98 |
| *ICAM1* | 1.05 (0.99 – 1.23) | 0.93 (0.75 – 1.02)^4^ | 0.93 (0.85 – 1.16) | 1.10 (0.84 – 1.18) | 0.047^3,5^ |
| *IL1RN* | 1.05 (0.86 – 1.16) | 0.97 (0.89 – 1.24) | 1.14 (0.83 – 1.36) | 1.04 (0.86 – 1.31) | 0.92^3^ |
| *IL1B* | 1.19 (0.76 - 1.26) | 0.80 (0.65 – 1.33) | 0.91 (0.76 – 1.52) | 1.10 (0.88 – 1.49) | 0.75 |
| *IL6* | 0.96 (0.60 – 2.40) | 1.02 (0.52 – 1.93) | 0.66 (0.56 – 1.10) | 0.94 (0.74 – 2.12) | 0.78 |
| *IL10* | 1.04 (0.77 – 1.39) | 0.85 (0.71 – 1.13) | 0.83 (0.59 – 1.52) | 1.14 (0.70 – 1.35) | 0.96 |
| *IL18* | 1.06 (0.76 – 1.18) | 1.09 (0.88 – 1.27) | 1.01 (0.77 – 1.24) | 0.99 (0.80 – 1.44) | 0.54 |
| *TNF* | 1.10 (0.90 – 1.34) | 0.91 (0.82 – 1.22) | 1.00 (0.81 – 1.14) | 1.03 (0.80 – 1.24) | 0.88 |
| *TNFRSF1A* | 0.95 (0.83 – 1.11) | 0.94 (0.82 – 1.05) | 1.05 (0.82 – 1.19) | 1.01 (0.86 – 1.19) | 0.87^3^ |
| *TNFRSF1B* | 1.15 (1.02 – 1.32) | 0.91 (0.82 – 0.99) | 1.06 (0.82 – 1.37) | 0.96 (0.79 – 1.21) | 0.24^3^ |
| *RELA* | 1.02 (0.96 – 1.19) | 0.89 (0.81 – 1.06) | 0.94 (0.87 – 1.02) | 0.95 (0.82 – 1.05) | 0.27 |
| *TLR2* | 0.95 (0.89 – 1.14) | 0.89 (0.66 – 1.07) | 1.22 (0.87 – 1.66) | 1.01 (0.85 – 1.31) | 0.09 |
| *TLR4* | 0.98 (0.88 – 1.23) | 0.92 (0.77 – 1.01) | 1.04 (0.86 – 1.39) | 1.14 (0.92 – 1.20) | 0.21^3^ |
| *IFNG* | 0.97 (0.74 – 1.11) | 0.91 (0.79 – 1.16) | 0.83 (0.69 – 0.98)^6^ | 1.08 (0.83 – 1.20) | 0.24 |

^1^ as Fold changes (FC): 12 week – 0 week. Group effect between fold changes adjusted for mRNA expression at 0 week (^2^ ANCOVA, ^3^ Quade’s test).

^4^ P=0.06 vs. fatty fish group after post-hoc pairwise comparisons with Bonferroni correction for multiple testing.

^5^ P=0.025 for the effect of lean fish group and P >0.10 for the effect of FF and CSO groups in relation to the control group in the ANCOVA model.

^6^ P=0.087 for the effect of the CSO and P >0.50 for the effect of FF and CSO groups in relation to the control group in the ANCOVA model.

**STable 5.** Changes^1^ in mRNA expression of immune-inflammatory-related genes from subcutaneous adipose tissue in each of the study groups (n = 39).

|  | Fatty fish, n = 8 | Lean fish, n = 10 | Camelina Sativa Oil, n = 9 | Control, n = 12 |  |
| --- | --- | --- | --- | --- | --- |
|  | FC^1^ (median (IQR)) | FC^1^ (median (IQR)) | FC^1^ (median (IQR)) | FC^1^ (median (IQR)) | P^2^ |
| *CCL2* | 0.79 (0.61 – 1.11) | 1.26 (0.81 – 1.42) | 1.28 (0.47 – 1.7) | 0.97 (0.78 – 1.07) | 0.67 |
| *ICAM1* | 1.06 (0.77 – 1.33) | 1.03 (0.82 – 1.17) | 1.07 (0.90 – 1.41) | 1.06 (0.82 – 1.39) | 0.97^3^ |
| *IL1RN* | 0.49 (0.29 – 0.96) | 0.89 (0.44 – 1.36) | 0.76 (0.45 – 1.21) | 1.64 (0.87 – 2.56) | 0.12^4^ |
| *IL1B* | 0.97 (0.54 – 1.76) | 0.81 (0.53 – 1.72) | 0.57 (0.45 – 0.91) | 0.91 (0.55 – 1.98) | 0.60 |
| *IL6* | 1.10 (0.47 – 1.77) | 1.10 (0.71 – 2.64) | 0.93 (0.75 – 1.51) | 0.79 (0.71 – 1.32) | 0.74^3^ |
| *IL10* | 0.85 (0.51 – 1.07) | 0.95 (0.71 – 2.22) | 0.96 (0.83 – 1.62) | 1.05 (0.65 – 1.61) | 0.63 |
| *TNF* | 1.17 (0.61 – 1.99) | 1.13 (0.80 – 1.34) | 0.78 (0.67 – 1.24) | 1.09 (0.87 – 1.49) | 0.31 |
| *TNFRSF1A* | 1.05 (0.83 – 1.40) | 0.98 (0.82 – 1.11) | 0.96 (0.87 – 0.99) | 0.89 (0.82 – 0.98) | 0.97^3^ |
| *TNFRSF1B* | 0.99 (0.61 – 1.99) | 0.99 (0.80 – 1.34) | 0.91 (0.67 – 1.24) | 0.91 (0.87 – 1.49) | 0.59 |
| *RELA* | 1.16 ± 0.30 | 1.02 ± 0.19 | 0.95 ± 0.19 | 0.93 ± 0.18 | 0.20 |
| *TLR2* | 1.19 (0.94 – 1.31) | 0.89 (0.76 – 1.25) | 0.97 (0.60 – 1.81) | 0.89 (0.73 – 1.15) | 0.94^3^ |
| *TLR4* | 1.14 (1.00 – 1.23) | 1.06 (0.95 – 1.39) | 1.06 (0.87 – 1.14) | 0.98 (0.88 – 1.16) | 0.34 |
| *ADIPOQ^5^* | 1.20 ± 0.35 | 0.97 ± 0.20 | 1.03 ± 0.37 | 0.95 ± 0.13 | 0.19 |

^1^ as Fold changes (FC): 12 week – 0 week.

^2,3^ Group effect between fold changes adjusted for mRNA expression at 0 week (^2^ANCOVA, ^3^ Quade’s test).

^4^ P=0.03 for the effect of fatty fish group and P>0.05 for the effect of LF and CSO groups in relation to the control group in the ANCOVA model.

**STable 6**. Correlations (ρ) of changes in *ICAM1* mRNA expression in PBMC and in *IL1RN* mRNA expression in SAT with the changes in plasma *n*-3 FAs in CE and phospholipid fractions

| Plasma *n*-3 FAs | *ICAM1* mRNA expression in PBMCs | | *IL1RN* mRNA expression in SAT |  |
| --- | --- | --- | --- | --- |
|  | All subjects, *n* = 72 | FF group, *n* = 19 | All subjects, *n* = 39 | |
| EPA in CEs | 0.15 | -0.19 | -0.32 | |
|  | *P* = 0.20 | *P* = 0.45 | *P* = 0.044 | |
| EPA in phospholipids | 0.12 | -0.21 | -0.17 | |
|  | *P* = 0.33 | *P* = 0.38 | *P* = 0.32 | |
| DHA in CEs | 0.00 | -0.26 | -0.19 | |
|  | *P* = 0.97 | *P* = 0.29 | *P* = 0.26 | |
| DHA in phospholipids | -0.06 | -0.40 | -0.09 | |
|  | *P* = 0.64 | *P* = 0.088 | *P* = 0.58 | |

CEs: cholesteryl esters. DHA: docosahexaenoic acid. EPA: eicosapentaenoic acid. FA: fatty acid.

FF: fatty fish. PBMCs: peripheral blood mononuclear cells. SAT: subcutaneous adipose tissue.
